# *HAMP* Downregulation Contributes to Aggressive Hepatocellular Carcinoma via Mechanism Mediated by Cyclin4-Dependent Kinase-1/STAT3 Pathway

**DOI:** 10.3390/diagnostics9020048

**Published:** 2019-04-30

**Authors:** Ying Shen, Xin Li, Yanwei Su, Shaikh Atik Badshah, Bin Zhang, Yanru Xue, Peng Shang

**Affiliations:** 1 Research & Development Institute of Northwestern Polytechnical University in Shenzhen, Shenzhen 518057, China; life-shenying@mail.nwpu.edu.cn (Y.S.); lixin5@mail.nwpu.edu.cn (X.L.); suyanwei@mail.nwpu.edu.cn (Y.S.); aatikshaikh@gmail.com (S.A.B.); hangbin_241@mail.nwpu.edu.cn (B.Z.); xueyanru@mail.nwpu.edu.cn (Y.X.); 2 School of Life Science, Northwestern Polytechnical University, Xi’an 710072, China; 3 Key Laboratory for Space Bioscience and Biotechnology, Institute of Special Environment Biophysics, School of Life Science, Northwestern Polytechnical University, Xi’an 710072, China

**Keywords:** hepatocellular carcinoma, *HAMP*, metastasis, cell cycle, iron

## Abstract

Background: Hepcidin encoded by *HAMP* is vital to regulating proliferation, metastasis, and migration. Hepcidin is secreted specifically by the liver. This study sought to examine the functional role of hepcidin in hepatocellular carcinoma (HCC). Methods: Data in the Cancer Genome Atlas database was used to analyze *HAMP* expression as it relates to HCC prognosis. We then used the 5-ethynyl-20-deoxyuridine (EdU) incorporation assay, transwell assay, and flow cytometric analysis, respectively, to assess proliferation, migration, and the cell cycle. Gene set enrichment analysis (GSEA) was used to find pathways affected by *HAMP*. Results: *HAMP* expression was lower in hepatocellular carcinoma samples compared with adjacent normal tissue controls. Low *HAMP* expression was linked with a higher rate of metastasis and poor disease-free status. Downregulation of *HAMP* induced SMMC-7721 and HepG-2 cell proliferation and promoted their migration. *HAMP* could affect the cell cycle pathway and Western blotting, confirming that reduced *HAMP* levels activated cyclin-dependent kinase-1/stat 3 pathway. Conclusion: Our findings indicate that *HAMP* functions as a tumor suppressor gene. The role of *HAMP* in cellular proliferation and metastasis is related to cell cycle checkpoints. *HAMP* could be considered as a diagnostic biomarker and targeted therapy in HCC.

## 1. Introduction

Hepatocellular carcinoma (HCC), as a cancer with one of the highest mortality rates, is a serious threat to public health [1]. Over the past 20 years, HCC-induced mortality has increased, and current estimates suggest it will continue to do so in the future. Roughly 85% of HCC occurs in developing nations and regions in Africa, Southeast Asia, and China, though rates are rising even in North America [2]. HCC incidence tripled in the United States from 1975 to 2005, and at present, roughly 20,000 cases are detected each year in the United States alone. Most patients are not diagnosed with HCC until they are in intermediate or advanced stages of the disease, at which point it is often impossible to cure [3]. Multiple therapeutic options are currently available for the treatment of HCC, and surgical removal of the tumor is probably the most common option. However, tumor metastasis is still the critical cause of HCC recurrence [4]. For these reasons, it is crucial to develop effective strategies to stratify tumors according to aggressiveness in the early stages.

The liver is the most important metabolism organ, and many bioprocesses such as energy metabolism, gene expression, and pathway signal transduction participate in hepatocellular carcinoma, including tumor proliferation, invasion, and metastasis [5,6,7]. Recently, a boom of studies has shown the role of iron in multiple cancers’ progression, invasion, migration, and death [8,9,10], as the liver is the most common iron metabolism and storage organ. Hence, the carcinogenesis and metastasis of hepatocellular carcinoma are highly related to iron [11]. A study reported that the expression of several iron metabolism pathways and iron transport genes is significantly altered in HCC relative to normal liver tissue, leading to an accumulation of intratumoral iron [12]. High iron concentration in cancer cells indicates more aggressive hepatocellular carcinoma [13]. Ferroptosis, a newly discovered type of cell death that is the result of high iron-concentration-dependent lipid peroxide accumulation, could contribute to HCC clinical therapy [14]. Above all, the role of iron metabolism in cancer cells and its implication in drug discovery and cancer therapy has aroused the attention of several scientists. 

The three main processes in cellular iron metabolism are iron intake, utilization, and efflux [15]. Iron is uptaken into the cells mainly when plasma transferrin binds to the transferrin receptor on the cell membrane [16]. The iron in transferrin is derived from food sources that are absorbed via the small intestine and from the breakdown of worn-out red blood cells by macrophages [17]. Ferroportin (FPN) protein is encoded by the *SLC40A1* gene and it is the most important iron efflux transporter, which pumps iron into the plasma of enterocytes and macrophages. The expression of FPN is regulated by hepcidin, which is a 25 amino acid peptide coded for by *HAMP* [18]. Though it was originally believed to be an antimicrobial peptide, hepcidin was instead soon determined to be a primary regulator of systemic iron metabolism and distribution via the hepcidin−ferroportin axis [19]. Hepcidin binds to FPN and causes its endocytosis and degradation, thereby preventing iron efflux into the plasma [15]. There is both experimental and epidemiologic evidence that dysregulated hepcidin–FPN signaling is linked to an elevated risk of hepatocellular carcinoma. In a fah^−/−^ mice model, hepatocytes lost the ability of transferrin-sensitive induction of hepcidin, thus inducing murine iron overload and liver injury [20]. Moreover, in alcoholic cirrhosis patients, low-serum hepcidin levels are associated with poor long-term survival [21]. As the key negative regulator of iron metabolism that is secreted primarily by the liver, low expression of hepcidin-induced hepatic iron overload can expedite the progression of liver diseases and the onset of HCC. However, the underlying mechanistic pathway by which low expression of hepcidin-induced HCC aggression and metastasis still remains unclear and requires further investigation.

It has been reported that high cellular iron levels could disturb the cell cycle in cancer cells by affecting the cyclin-dependent kinases activity [22]. The STAT3 pathway is one of the most important pathways that has been shown to influence both changes in the cell cycle as well as *HAMP* expression [15]. In this study, we assessed the effects of *HAMP* expression on the cell cycle of HCC cells and further explored whether the STAT3 pathway could play a role in *HAMP* downregulation-induced aggressive HCC by knockdown of *HAMP*.

In summary, cellular *HAMP* expression is commonly dysregulated in HCC. In this study, we analyzed the expression of *HAMP* and *SLC40A1* in an HCC cohort from The Cancer Genome Atlas (TCGA) and validated the results by experiments in vivo and in vitro. In addition, we assessed the prognosis of HCC patients based on TCGA database according to *HAMP* expression. Using *HAMP* shRNA to downregulated *HAMP* expression, we explored the mechanisms of *HAMP*-induced aggressive HCC.

## 2. Materials and Methods

### 2.1. Patients and Samples

TCGA was the source of all clinical and related gene expression data, using the HCC cohort in the Genomic Data Common (GDC) data portal. We were able to obtain clinical and RNA-seq data from 423 HCC patients. We defined genetic alterations as gene mRNA high expression if greater than the medium value [23].

### 2.2. Cell Culture

DMEM containing 10% FBS as well as penicillin/streptomycin was used to culture HepG-2 and SMMC-7721 cells (cell lines obtained from ATCC). For individual experiments, 100,000 cells were plated per well on a six-well plate for 12 h unless otherwise indicated.

### 2.3. Western Blotting

We plated 250,000 cells/well on a six-well plate and transfected them as indicated. A mammalian lysis buffer with protease inhibitor cocktail (Beyotime, Shanghai, China) was then used to lyse cells. Extracted protein was electrophoretically separated using 10%–15% SDS-PAGE gels and then transferred to PVDF membranes. These membranes were then probed using anti-*HAMP* (1:1000; Abcam, Cambridge, UK, Rabbit AB75883), anti-GAPDH (1:5000; Proteintech, Mouse AB8226), anti-CDK1(1:1000; Invitrogen, Carlsbad, CA, USA, H68.4), and anti-STAT3 (1:1000; Abcam, Rabbit AB75883). Next, HRP-labeled secondary antibodies were used to detect primary antibodies, and an ECL plus Detection System (T5200, Tanon) was used to visualize proteins, with densitometry being calculated via Image J.

### 2.4. HAMP shRNA Transfection

*HAMP* Silencer shRNA (Genechem, Shanghai, China, sequence: (5′–3′) CGCTTGCCTCCTGCTCCTCCT; antisense AGGAGGAGCAGGAGGCAAGCG) and *HAMP* overexpression vector (Genechem, sequence: (5′–3′) GAGGATCCCCGGGTACCGGTCGCCACCATGGCGGAGCCGAGCGGC; antisense TCACCATGGTGGCGACCGGGCTGACACTCAACTGAGCA) were prepared at a stock 100 concentration, from which working 10-µM stocks were prepared for each individual use. Opti-DMEM medium (Gibco, Life Technologies, Waltham, MA, USA) was used to dilute Lipofectamine 3000, and then shRNA was added at a final concentration of 20 pM of shRNA for 10 min. The combined mixture was then added onto target cells, which were collected 48 h later. As a negative control, cells were also transfected using Silencer Select Negative Control shRNA, as mentioned above.

### 2.5. Quantitative RT-PCR

TRIzol (Invitrogen, Carlsbad, CA, USA) was used to extract total RNA based on provided protocols. This RNA was then reverse transcribed to cDNA via a PrimeScript RT kit (TaKaRa, Beijing, China). cDNA was next used for qPCR with SYBR Premix Ex Taq (TaKaRa) in a CFX96 Touch qPCR System (Bio-Rad Laboratories, Hercules, CA, USA). Table 1 describes all the primers used, and gene expression was normalized to GAPDH via the 2^−ΔΔ*C*t^ approach [24].

### 2.6. Tumor Colony Forming Assay

For colony forming assays, we plated 1000 HepG-2 and SMMC-7721 cells per well on six-well plates overnight and then transfected these cells with *HAMP* shRNA or control. After 24 h, media was changed, and 10 days later, we assessed cells to count the colonies formed. Media were changed every two days during this time. On day 10, cells were fixed for 20 min using 4% paraformaldehyde, stained via crystal violet (Sigma-Aldrich, San Francisco, CA, USA) and imaged to count colonies.

### 2.7. 5-Ethynyl-20-deoxyuridine (EdU) Incorporation Assay

Cells were labeled with EdU based on provided directions (RiboBio, Guangzhou, China). Briefly, we plated 250,000 HepG-2 and SMMC-7721 cells per well on a six-well plate overnight. Cells were transfected with *HAMP* shRNA and control *HAMP* shRNA as the vehicle. Twenty-four hours post-transfection, fresh the culture medium was added and further incubated for another 24 h. Then, cells were tested by an EdU staining kit as per the protocol. SMMC-7721/HepG-2 cell staining was observed under a fluorescent inverted microscope (Leica DMIL, Leica Microsystems, Solms, Germany).

### 2.8. Wound-Healing Assay

To measure migration, we plated 1,000,000 stably transfected control vector or sh-*HAMP* SMMC-7721 cells and HepG-2 cells per well on six-well plates in a 2-mL volume for 24 h. We then generated a wound in the cell layer using a 200-µL tip, and cells in this area were removed via PBS rinsing. Low-serum media were then added to the cells (DMEM + 0.5% FBS), and a Leica DMIL inverted microscope was used to image cells at 0 and 24 h. The area of this wound was measured in Image J, and the degree of wound closure was calculated as follows: wound closure = wound area at 0 h − wound area at 24 h.

### 2.9. Cell Cycle Analysis

We plated cells at 2,000,000/mL in six-well plates and then transfected them using control vector or sh-*HAMP* SMMC-7721 for 24 h. We then collected cells, washed them using PBS, and fixed them overnight using 75% ethyl alcohol at 4 °C. After washing twice in PBS, cells were treated for 30 min with RNAse A and were then stained using 500 µL of propidium iodide at room temperature for 30 min. An Accuri C6 (BD Biosciences, New York, NY, USA) was then used for cell cycle analysis.

### 2.10. Animal Treatment

The SPF Animal Laboratory of Beijing Sibeifu approved this study (animal authorization reference number: SCXK2016-0002, approval date: 30 August 2016). Male BALB/c-nu mice were 4-weeks old and housed under standard conditions with free food/water access. The animals were cared for in accordance with the institution’s guidelines. Mice were randomized into two groups: the control group and the *HAMP* shRNA1 groups (five mice in each group). Control or *HAMP*-shRNA-transfected SMMC-7721 cells were digested, washed thrice in PBS, and adjusted to 10,000,000 cells/mL in cold PBS. A total of 100 μL of this suspension was then injected subcutaneously. After 4 weeks, mice were sacrificed and tumors were isolated and weighed.

### 2.11. Statistical Analysis

Data are means and standard deviations. Results were compared via paired *t*-tests or ANOVAs.

## 3. Results

### 3.1. HAMP Expression Is Significantly Suppressed in Hepatocellular Carcinoma

**Figure 1:**
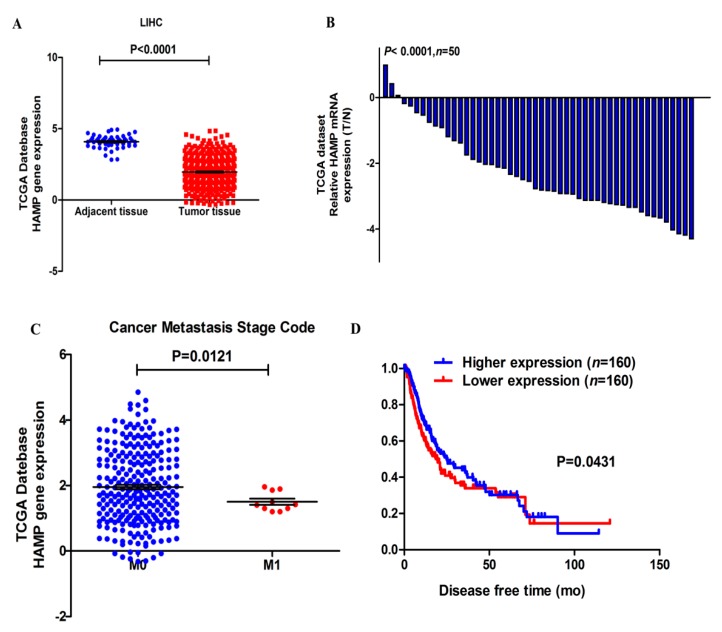
*HAMP* expression is reduced in liver hepatocellular carcinoma (HCC) and low *HAMP* expression is linked to higher cancer metastasis stage code and poor disease-free status. (**A**) *HAMP* expression in HCC tumors was significantly decreased relative to adjacent liver tissue (*p* < 0.0001). (**B**) Histogram showing *HAMP* expression in liver hepatocellular cancer. The 2^−ΔΔ*C*t^ approach was used to calculate *HAMP* expression, and expression in each patient is given as the tumor (T, *n* = 50)/normal (N, *n* = 50) ratio; (**C**) Low *HAMP* expression is associated with high cancer metastasis in HCC patients (*p* = 0.0121). (**D**) Low *HAMP* expression is associated with poor disease-free status in HCC patients, according to a Kaplan–Meier analysis of HCC (*n* = 320, log rank test, *p* = 0.0431).

The expression of *HAMP* was assessed in 423 samples, including 373 tumor tissue samples and 50 adjacent tissue samples from TCGA dataset. *HAMP* expression was significantly inhibited in liver tumor tissues compared with adjacent tissue (Figure 1A). In addition, the mean folds change of *HAMP* in tumor tissues was about 230.3 compared with adjacent tissues (Figure 1B). These data showed *HAMP* was significantly inhibited in HCC.

### 3.2. Lower HAMP Expression Is Associated with Higher Cancer Metastasis Rate and Recurrence

We first evaluated TCGA data to assess if *HAMP* was associated with important outcomes in HCC. Our results showed that HCC patients with low *HAMP* expression had poorer outcomes. (Table 1). This dataset contains specimens from 127 patients diagnosed with HCC and annotated with cancer metastasis and disease-free status data. Analysis of *HAMP* expression of HCC tissues with M0 or M1 cancer metastasis grade showed that tumor tissues with higher metastasis grade had lower *HAMP* expression (Figure 1C). Furthermore, tumor tissues with low *HAMP* expression were linked to a significantly worse disease-free status (Figure 1D). These results suggest that lower *HAMP* expression could induce poorer prognosis in HCC patients.

### 3.3. Low Expression of HAMP Results in Increased Proliferation and Migration in SMMC-7721 and HepG-2 Cells

**Figure 2:**
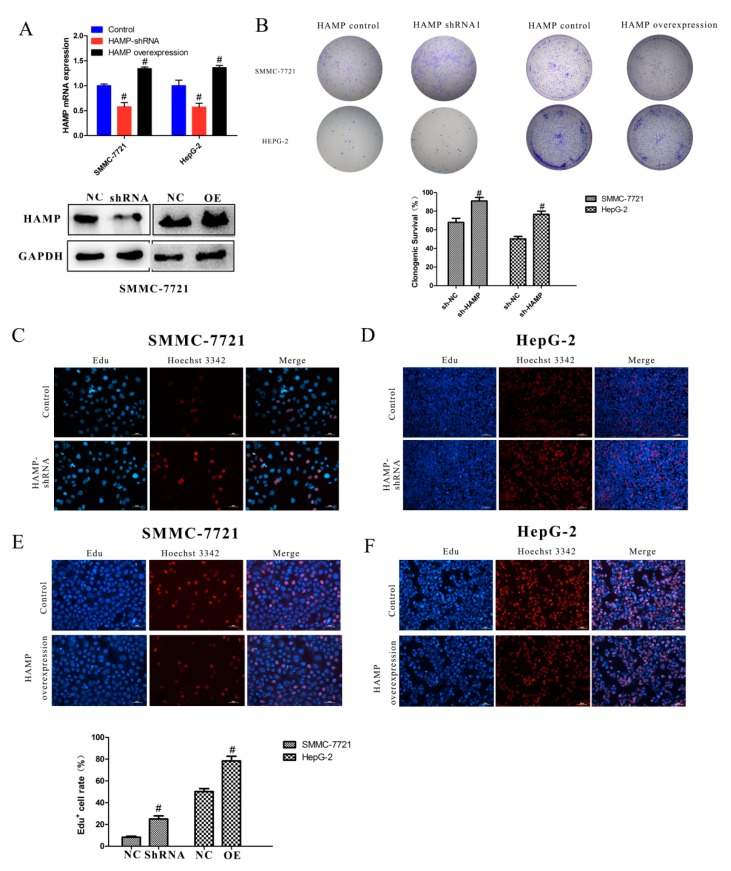
The effect of *HAMP* on proliferation. (**A**) Analysis of *HAMP* expression in SMMC-7721/HepG-2 cells after transfection with control, *HAMP* shRNA, and *HAMP* overexpression vector. (**B**) A tumor colony forming assay was used to detect the proliferation of SMMC-7721 and HepG-2 cells after knockdown or overexpressed the *HAMP* gene expression. (**C**–**F**) 5-ethynyl-20-deoxyuridine (EdU) incorporation assay was employed to detect the viability of SMMC-7721/HepG-2 cells transfected with control, *HAMP* shRNA, and *HAMP* overexpression vector. ^#^ *p* < 0.05. Scale bar = 50 µM

**Figure 3:**
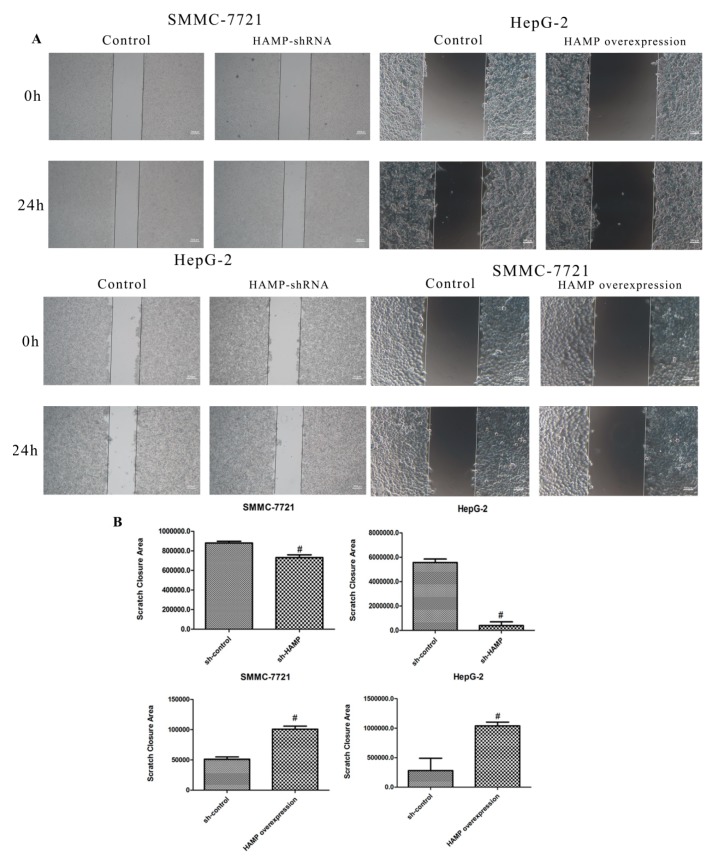
The effect of *HAMP* on the migration of liver cancer cells. (**A**,**B**) Migration was measured via wound-healing assay for SMMC-7721/HepG-2 cells transfected with control, *HAMP* shRNA, and *HAMP* overexpression vector. ^#^ *p* < 0.05. Scale bar = 300 µM

Our study revealed that a majority of HCC tissues showed reduced expression of *HAMP* expression. To determine the effect of *HAMP* low expression on phenotypic properties in hepatocellular carcinoma cells, we used *HAMP shRNA* to knockdown *HAMP* expression and *HAMP* overexpression vector to induce *HAMP* overexpression. *HAMP* expression in SMMC-7721 and HepG-2 was significantly inhibited by *HAMP shRNA1* transfection and was significantly increased by *HAMP* overexpression vector transfection by using RT-PCR and Western blot analysis (Figure 2A). To determine the effect of *HAMP* expression on proliferation of SMMC-7721 and HepG-2, we used colony forming assays. The analysis shows that low *HAMP* expression significantly promoted the proliferation of SMMC-7721 and HepG-2 cells and high *HAMP* expression inhibited the proliferation of SMMC-7721 and HepG2 cells (Figure 2B). To further confirm the effect of *HAMP* expression on cell proliferation, an EdU incorporation assay had to be carried out. Our data showed that knockdown of *HAMP* significantly increased the number of EdU-positive cells, while overexpression of *HAMP* inhibited the number of EdU-positive cells compared with the control group in SMMC-7721 and HepG-2 cells (Figure 2C–F). Additionally, TCGA analysis demonstrated that hepatocellular carcinoma in patients with lower *HAMP* expression had a higher disease recurrence rate and metastasis grade. We assumed that downregulation of *HAMP* could increase the hepatocellular carcinoma invasiveness. A wound-healing test of HepG-2 cells and SMMC-7721 with *HAMP* shRNA transfection showed that lower expression of *HAMP* increased the SMMC-7721 and HepG-2 cell migration ability, while higher expression of *HAMP* decreased the SMMC-7721 and HepG-2 cell migration ability (Figure 3A–B). These data indicate that low *HAMP* expression promotes the proliferation and migration of human hepatocellular carcinoma.

### 3.4. Low HAMP Expression Promotes Hepatocellular Carcinoma Proliferation In Vivo

**Figure 4:**
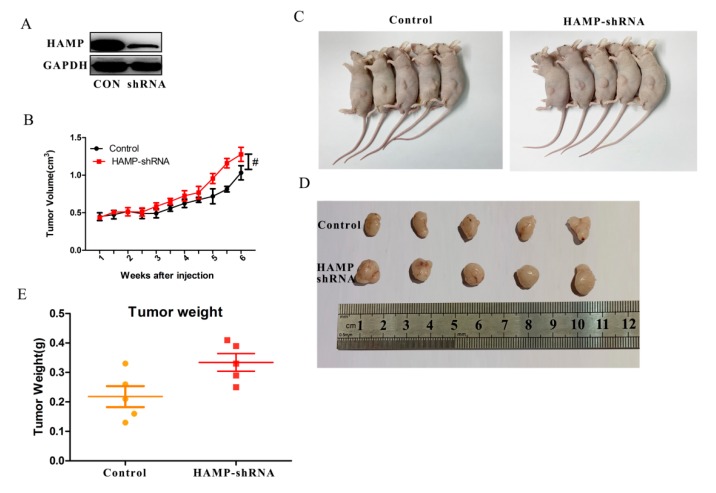
*HAMP* affects hepatocellular carcinoma proliferation in vivo. SMMC-7721 cells transfected with control or shRNA were injected s.c. in BALB/c-nu mice. (**A**) *HAMP* expression in tumors. (**B**) Tumor volume, measured every other day. (**C**,**D**) Tumor volumes changes in control and shRNA mice. (**E**) Tumor weight changes in control and shRNA mice. (*n* = 5). ^#^ *p* < 0.05.

In order to analyze the effect of *HAMP* on hepatocellular carcinoma in vivo, we used nude mice bearing tumor experiment. SMMC-7721 cells transfected with control or *HAMP* silencer shRNA were injected subcutaneously. After 4 weeks, the mice were sacrificed, and the tumors were removed, weighted. Figure 4A–E showed that low expression of *HAMP* promoted tumor volume growth. In *HAMP* shRNA groups, tumor weights were significantly higher than control groups. These data showed that low expression of *HAMP* promotes hepatocellular carcinoma proliferation in vivo.

### 3.5. Low HAMP Expression Is Associated with Cell Cycle Checkpoint in Gene Set Enrichment Analysis (GSEA) of the TCGA

**Figure 5:**
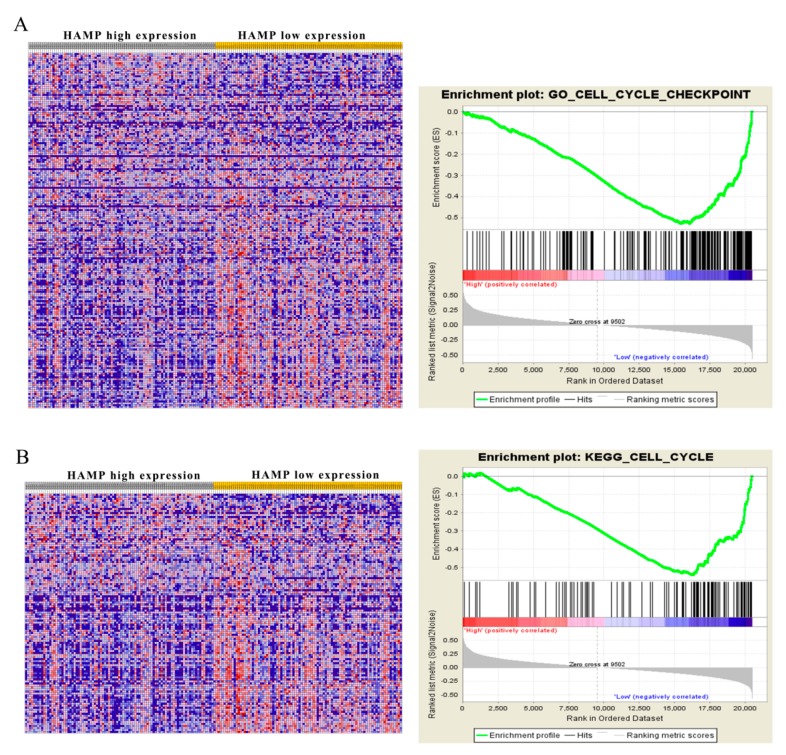
Gene set enrichment analysis (GSEA) based on *HAMP* expression in HCC patients. To identify pathways linked to *HAMP* expression in The Cancer Genome Atlas (TCGA) samples, we compared gene expression in those with low *HAMP* expression (blue) and high *HAMP* expression (red). Visualizations were produced using Cytoscape and Enrichment map (1% FDR, *p* < 0.005). Each node is representative of a set of enriched genes, with annotations being ascribed based on how similar the sets are to one another. A network of nodes was used to map the enrichment results, with the size of nodes being directly correlated with the gene number in that particular gene set. The number of genes shared between two given gene sets determined the thickness of the green line connecting them. The final map was generated via removal of any general or noninformative smaller networks in order to simplify the final diagram. (**A**) Go enrichment plot showed *HAMP* downregulation is related to cell cycle checkpoint. (**B**) KEGG enrichment plot showed *HAMP* downregulation is related to the cell cycle.

Given that low expression of *HAMP* accelerated the proliferation and migration of hepatocellular carcinoma cell, we used the Gene Set Analysis of the TCGA to explore potential *HAMP* targets. We divided the data of the TCGA into *HAMP* lower expression and *HAMP* higher expression according to the median *HAMP* expression in TCGA dataset. Comparison of transcript levels in *HAMP*-high and -low tumors revealed a range of differentially expressed genes. Our data showed *HAMP* expression significantly affected the cell cycle checkpoint and associated with the cell cycle of hepatocellular carcinoma (Figure 5A,B). GSEA without gene parsing also identified G2M checkpoint targets as being highly enriched. Collectively, GSEA analysis of TCGA indicated that low *HAMP* expression affects the activation of the G2M checkpoint to influence the cell cycle of the hepatocellular carcinoma, which contributes the aggressive HCC.

### 3.6. HAMP Suppression Results in High Cellular Iron Concentration in Cells and Activated CDK1/STAT3 Pathway

**Figure 6:**
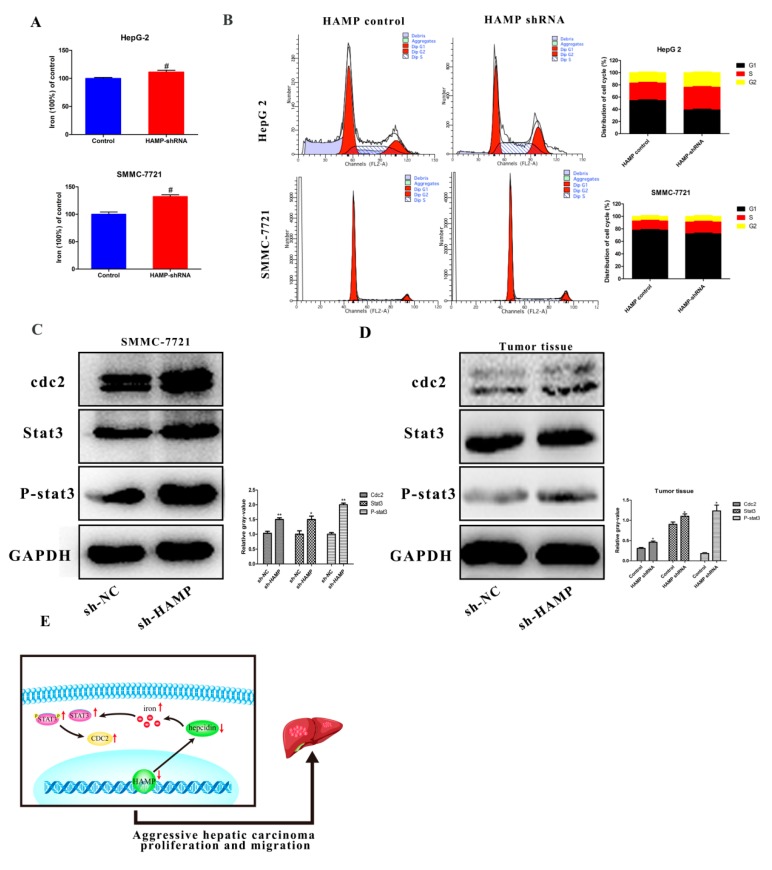
*HAMP* downregulation activates the cdk1/stat3 pathway. (**A**) Knockdown of *HAMP* promotes cellular iron concentration in SMMC-7721/HepG-2 cells. (**B**) Cell cycle analysis of SMMC-7721 and HepG-2 cells transfected with *HAMP* shRNA. (**C**,**D**) *HAMP* downregulation activates the cdk1/stat3 pathway. (**E**) Downregulation of *HAMP* low expression of hepcidin, which promotes cellular iron concentration and then activates the cdk1/stat3 pathway to promote tumor proliferation and metastasis. ^#^ *p* < 0.05, * *p* < 0.05 and ** *p* < 0.01.

Hepcidin encoded by the *HAMP* gene is one of the most critical molecular regulators for cellular and systemic iron metabolism. In this study, we tested the iron concentration in SMMC-7721 and HepG-2 cells transfected with *HAMP* shRNA to determine the effects of hepcidin iron metabolism. Our data showed that downregulation of *HAMP* could increase cellular iron concentration (Figure 6A). It was reported that iron contributes to multiple bioprocesses, including DNA synthesis, enzyme catalysis, redox metabolism, cell cycle regulation, and multiple signaling pathways. In Figure 5, GSEA analysis shows that low *HAMP* expression affected the cell cycle pathway. Furthermore, low expression of *HAMP* increased the cellular iron concentration. We hypothesized that the increase in cellular iron concentration may activate cell cycle checkpoints and related pathways. In Figure 6B, we found that low expression of *HAMP* induces the proportion of cells in the S phase of the cell cycle. As cyclin-dependent kinases 1/stat3 was reported as a critical pathway affected by iron concentration in tumor proliferation and migration, we tested the expression of the cyclin-dependent kinases 1/stat3 pathway. Western blotting was performed to assess changes in the expression of cdk1/stat3-related molecules in the SMMC-7721 cells transfected with either control vector or shRNA *HAMP*. The results showed that after knockdown of the *HAMP* expression in SMMC-7721 cells and tumor tissues, the expression of cyclin-dependent kinases 1(cdc2) was significantly increased, and the expressions of stat3 and phospho-stat3 were significantly increased (Figure 6C,D), respectively. These findings suggest that downregulation of *HAMP* could activate the cdk1/stat3 pathway.

In summary, our study showed that the expression of *HAMP* was downregulated in tumor tissues compared with adjacent tumor tissues in HCC patients. Furthermore, HCC patients with lower expression of *HAMP* showed a higher rate of metastasis and recurrence rate. *ShRNA HAMP* was used to knockdown the *HAMP* expression in SMMC-7721 and HepG-2 cells. The cell proliferation and migration assay showed that the downregulation of *HAMP* resulted in increased cell proliferation and migration. GSEA analysis showed that the downregulation of *HAMP* activated the cell-cycle-related pathways to induce cell proliferation and migration. The Western blotting test showed low expression activated the cdk1/stat3 pathway. These findings, thus, suggest that the downexpression of *HAMP* could induce HCC proliferation and migration via activating the cdk1/stat3 pathway (Figure 6E).

## 4. Discussion

Iron is one of the most crucial elements in humans for its indispensable biological functions in life. Iron intake in the human body includes endogenous and exogenous iron intake. Endogenous iron is derived from macrophages which engulf and digest aging red blood cells and then release iron through the FPN channel. Exogenous iron intake relies on FPN channels that absorb iron from digested food through intestinal epithelial cells. Together, endogenous and exogenous iron maintain systemic iron in a dynamic equilibrium. Systemic iron metabolism is regulated by the hepcidin–ferroportin axis, while hepcidin plays a key role in iron homeostasis. A high level of systemic iron can promote the expression of hepcidin, which binds with the FPN, thus inducing FPN endocytotic degradation, while a low level of systemic iron inhibits the expression of hepcidin and activates the FPN. Dysregulated hepcidin-expression-induced iron metabolism imbalance is related to multiple diseases, including anemia [25], Gaucher disease [26], neurodegenerative diseases [27], and cancer [28,29].

Tumor cells require large amounts of intracellular iron to supply cell proliferation, migration, and invasion. Hepcidin expression in tumor cells is strongly correlated with regulation within tumor cells relative to normal cells. Hepcidin expression is substantially elevated in colorectal cancer, whereas it is largely absent in nearby normal tissues [30]. Similarly, in prostate cancer, hepcidin is highly expressed in cancer cells and linked to regulating cell proliferation, migration, and apoptosis by increasing intracellular iron transportation [31]. Moreover, in breast cancer, there were some limited increases in hepcidin expression within tumors relative to nearby normal tissues [32]. Chen et al. found that the serum hepcidin concentration was higher in patients with NSCLC than in noncancerous individuals [33]. In these cancers, increased serum hepcidin mainly allows for increased iron within tumor cells, given that higher levels of local hepcidin result in the degradation of FPN, thus preventing iron export and increasing iron levels within tumor cells to facilitate survival and proliferation [34].

However, the expression of hepcidin showed the opposite result in liver cancer [35]. In HCC, levels of hepcidin are lower in tumor samples than in controls, unlike in other tumor types [36]. This phenomenon may be regulated by unique molecular pathways in the liver. A few studies suggest that BMP6 and HJV, which are inhibited in liver cancer, are the main regulator of hepcidin expression [37,38], while studies have also shown that the low expression of hepcidin predicts poor prognosis in liver cancer patients. Nahon et al. assessed how baseline hepcidin levels in the serum influenced the outcomes in 237 patients suffering from alcoholic cirrhosis. Their study determined that lower levels of serum hepcidin were linked to poorer long-term survival [21]. 

## 5. Conclusions

Our study found that *HAMP* is downregulated in liver cancer and indicates poor disease prognosis. As showed above, *HAMP* in liver cancer is regulated by a specific pathway to satisfy tumor proliferation and migration. Our results also showed that the cancer cells with low hepcidin had a higher cellular iron level, which can activate cell cycle checkpoints and promote cancer cell proliferation and migration. Overall, our study suggests that *HAMP* could be a potential diagnostic and therapeutic target for the treatment of HCC.

## Figures and Tables

**Table 1:** Clinical characteristics according to *HAMP* expression in liver hepatocellular cancers.

Pathology Character	*n*	*HAMP* Expression		*p* Value
		Low	High	
Adjacent inflammation				0.3829
None	117	63	54	
Mild	100	46	54	
Severe	17	6	11	
M stage				0.0121 *
M0	267	135	132	
M1	4	0	4	
T stage				0.0361 *
T1	181	82	99	
T2	94	48	46	
T3	80	47	33	
T4	13	7	6	
Age (year)				0.1581
≤60	177	81	96	
>60	195	104	91	
Postoperative radiotherapy				0.1339
No	241	121	120	
Yes	4	2	2	
Family history				0.8110
No	208	100	108	
Yes	112	60	52	
History risk factor				0.1123
Alcohol consumption	68	37	31	
Hemochromatosis	6	4	2	
Hepatitis B	76	37	39	
Hepatitis C	32	13	19	
Liver fibrosis Ishak score				0.6397
0—No Fibrosis	74	40	34	
1,2—Portal Fibrosis	31	16	15	
3,4—Fibrous Speta	28	14	14	
5—Nodular Formation	9	3	6	
6—Established Cirrhosis	70	35	35	
Lymph node stage				0.4924
N0	253	125	128	
N1	4	3	1	
Neoplasm stage				0.9142
Stage I	171	78	93	
Stage II	86	43	43	
Stage III	85	52	33	
Stage IV	5	1	4	
Neoplasm histologic grade				0.0129 *
G1	55	19	36	
G2	178	71	107	
G3	122	40	82	
G4	12	3	9	
Sex				0.4953
Female	121	91	30	
Male	250	192	58	

* *p* < 0.05.

## Abbreviations

CDK1	Cyclin-dependent kinases 1
FPN	Ferroportin
GSEA	Gene Set Enrichment Analysis
*HAMP*	Hepcidin
HCC	Hepatocellular carcinoma
*SLC40A1*	Solute carrier family 40 member 1
STAT3	Signal transducer and activator of transcription 3
TCGA	The Cancer Genome Atlas
